# Atlas to the Pathology of the Teeth

**Published:** 1869-05

**Authors:** 


### BIBLIOGRAPHICAL NOTICES.

*Atlas zur Pathologie der Zahne.* Bearbeitet von Weil. Prof. Dr. M. Heider und Prof. Dr. C. Wedl. Die Zeichmungen Sammtlich nach der natur aufgenommen von D. C. Heitzmann. Leipsic, London and New York.

Two numbers of this Atlas to the Pathology of the Teeth, arranged and explained by the late Prof. Dr. M. Heider and Prof. Dr. C. Wedl, with drawings from nature by Dr. C. Heitzmann, have been issued, which are the first, we understand, of a text book on dental pathology.

The two numbers contain eight folio pages and eighty-one well executed lithographs of preparations from the pathological collections of Prof. Heider. Two more numbers complete the work which will prove a valuable one to every dentist and dental student.

Prof. Heider was for many years a prominent instructor in the Vienna University, and Prof. Wedl, who has contributed valuable monograms to science, has arranged the atlas and text, which is in both English and German, from the valuable collections left by Prof. Heider.

A work of this kind will supply a want long felt, as no complete one devoted wholly to dental pathology exists.

